# Association between depression and health care expenditure in middle-aged and older patients with heart disease

**DOI:** 10.1371/journal.pone.0328491

**Published:** 2025-07-17

**Authors:** Shushu You, Xiang Gu, Jing Sui, Ziyu Xiang, Fan Gao

**Affiliations:** 1 School of Law and Public Affairs, Research Institute for Environment and Health, Nanjing University of Information Science and Technology, Nanjing, China; 2 School of Public Health, Shandong University, Jinan, China; 3 Institute of Public Safety Governance, School of Emergency Management, Nanjing University of Information Science & Technology, Nanjing, China; 4 Key Laboratory of Environmental Medicine Engineering, Ministry of Education, School of Public Health, Southeast University, Nanjing, China; 5 Business School of Hohai University, Hohai University, Nanjing, China; University of Florida, UNITED STATES OF AMERICA

## Abstract

**Objective:**

Patients with heart disease frequently experience a heightened incidence of depression, thereby affecting their overall medical expenditures. Nevertheless, earlier investigations have focused on the healthcare costs incurred by individual patients with heart disease or those with depression. The present study assessed the correlation between depression and the medical expenditures of patients with heart disease, highlighting the importance of mental health care for patients with with heart disease.

**Method:**

Using data from Charles, we identified 2445 middle-aged and older patients with heart disease based on their completed medical expenditure-related questionnaires. The study further calculated the questionnaire data and used multiple linear regression analysis to control for demographic variables to evaluate the relationship between depressive symptoms and medical expenses.

**Results:**

In our sample, the presence or absence of depressive symptoms exhibited statistically significant differences (p < 0.05) in the medical expenditures of patients. Approximately 50.27% of heart disease patients had depressive symptoms. Depression level, gender, education level, type of health insurance, consumption level, and number of other chronic diseases were significantly associated with higher medical expenditures. A positive correlation was found between depressive symptoms and medical expenses.

**Conclusion:**

Heart disease patients exhibiting depressive symptoms incurred higher medical expenditures compared to their counterparts without depression. The findings of this study imply the need to augment mental health care services for older individuals with heart disease and to enhance collaborative care interventions within the context of heart disease.

## Introduction

In recent times, chronic diseases have emerged as the predominant contributor to healthcare expenditures [1]. Heart disease—a prominent non-communicable chronic disease—imposes a substantial financial burden on both affected individuals and the entire nation. In 2017–2018, cardiovascular disease accounted for 12% of the overall health expenditures in the United States [2]. In 2019, the per capita medical expenditure for older patients afflicted with heart disease amounted to $5,397, ranking second among the nine commonly treated conditions [3]. Meanwhile, the prevalence and mortality rates of heart disease exhibit an upward trend with advancing age. Based on the findings of a cross-sectional national survey, it is anticipated that the older population of China will experience a substantial rise in the incidence of degenerative heart disease in the coming years [4]. Meanwhile, there is a noticeable trend of heart disease occurring at younger ages [5]. In general, the prevalence of heart disease in the entire Chinese population is showing a consistent upward trend [6]. Heart disease-related fatalities in China accounted for approximately 40% of all disease-related deaths in 2018, and this figure will increase steadily in the future [7]. Given the increasing prevalence and mortality rates, heart disease-related medical expenditures will pose a serious challenge to public health and the Chinese healthcare system.

Heart disease often has high rates of depressive symptoms [8], and older patients are at higher risk for depression because of physiological differences and lower socioeconomic status. The incidence rates of anxiety and depression disorders among individuals with cardiovascular system diseases range from 13% to 28% [9]. Approximately 21.5% of patients with heart failure exhibit clinically significant depressive symptoms [10], while almost two-thirds of those experiencing acute myocardial infarction manifest such symptoms. Furthermore, individuals with infarction are prone to developing mild depression, and approximately 30% of stroke patients experience depression during either the early or late stages of the disease [11]. As a cardiovascular disease risk factor, depressive symptoms can aggravate the condition of individuals with heart disease. These symptoms may persist in patients for months following the acute phase of heart disease, contributing to an extended period of heightened morbidity [12] and influencing subsequent decisions regarding treatment regimens and the utilization of healthcare services. A study in China reported that patients with coronary heart disease who suffer from depression often have difficulty obtaining good social support, thereby heightening the probability of subsequent adverse cardiac events [13]. A cohort study conducted in the United States revealed that patients with atherosclerotic cardiovascular disease (ASCVD) who were at high risk for depression demonstrated a propensity for non-compliance with recommended secondary preventive measures. The patients also exhibited challenges in effective communication with healthcare providers, thereby affecting treatment outcomes as well as both physical and mental health [14]. Furthermore, patients with acute coronary syndrome (ACS) experiencing comorbid depressive and anxiety symptoms exhibited a notably higher frequency of re-hospitalization and emergency room visits compared with the general ACS patient population [15].

Studies have explored the relationship between depressive symptoms and heart disease [16]. However, their primary emphasis remains on investigating the influence of depressive symptoms on morbidity, mortality, and quality of life among individuals with heart disease. A 5-year retrospective cohort study conducted in South Korea tracked heart disease patients newly diagnosed with depression and demonstrated that depression increased the risk of ischemic heart disease and cerebrovascular disease in older Koreans by 38% and 46%, respectively [17]. A study conducted in China demonstrated that depressive symptoms worsened the condition of individuals with heart disease and that heart disease patients with comorbid depression had a significantly higher mortality rate than those without depression [18]. Another study on the effect of depression on sleep quality in patients with coronary artery disease showed a significant association between sleep disturbances and both depression and anxiety. This association further contributed to a notable decrease in the overall quality of life for patients with coronary artery disease [19]. Furthermore, studies have discussed the economic costs associated with depression and heart disease. A meta-analysis examining studies on the excess costs of depression found that these costs tend to rise when depression coexists with other illnesses [20]. However, the analysis did not explicitly discuss additional medical costs associated with depression in patients with heart disease. A cross-sectional study conducted in Turkey confirmed the substantial economic burden associated with heart failure, including both direct and indirect costs [21]. However, there is currently limited research on the financial costs associated with the confluence of depression and heart disease. The present study explores the association between depressive symptoms and medical costs in patients with heart disease,offering insights that can serve as a reference for patients’ subsequent medical care services.

## Methods

### Patients and data sources

The data for this study were derived from the 2018 China Health and Retirement Longitudinal Study (CHARLS, https://charls.pku.edu.cn/). In 2018, the survey used a stratified multi-stage (by GDP per capita in urban and rural districts) PPS random sampling strategy targeting middle-aged and older adults aged 45 years and above. The survey covered 150 countries/districts, spanning 450 villages and urban communities nationwide, involving 17,708 individuals across 10,257 households [22], reflecting the overall situation of middle-aged and older Chinese people. The present study received ethical approval from the Peking University Biomedical Ethics Committee.

The data from the fourth CHARLS survey, which surveyed 17,708 people, was analyzed in the present study. Whether people had heart disease was judged based on the question: Have you been diagnosed with heart attack, coronary heart disease, angina, congestive heart failure, or other heart problems by a doctor? The study excluded invalid samples, such as patients with non-cardiac disease who refused to answer or incomplete surveys. Finally, the study included 2445 participants.

### Criteria for depressive symptoms

The study used the 10 question version of the CES-D to assess depressive symptoms. The scale contains ten questions, and the response options are divided into four levels: rarely or none of the time = 0; some or a little of the time = 1; occasionally or a moderate amount of the time = 2; most or all the time = 3. A score of 10 or higher on a scale of 0–30 indicates the presence of depressive symptoms.

### Medical utilization and expenditures

The annual medical expenditure data for older patients with heart disease in this study are derived from the sum of outpatient medical expenditures, self-medication expenditures, and inpatient medical expenditures in the past year in CHARLS data. Among them, outpatient medical expenditure is derived from the question in the CHARLS questionnaire – How much did all the visits to medical facilities spend during the last month? (Include self-paid part and reimbursement part)? Annual outpatient medical expenditures are calculated by multiplying the amount by 12. Self-medication expenditures are derived from the following item in the CHARLS questionnaire: What is the approximate total cost for purchased medicine during the last month? (Include out-of-pocket part and reimbursement part). Annual outpatient medical expenditures are calculated by multiplying the amount by 12. The hospitalization medical expenditure is derived from the following item in the CHARLS questionnaire: What was the total medical cost for all the inpatient care you received during the past year? (Include the out-of-pocket part and reimbursement part. Only include fees paid to the hospital, including ward fees but excluding wages paid to a hired nurse, transportation costs, and accommodation costs for yourself or family members) The three values are added together to obtain the total medical expenditures of older patients with heart disease.

### Research variables

The healthcare expenditures of middle-aged and older cardiac patients served as the dependent variable. Other research variables include the number of chronic diseases (diabetes, stroke, Alzheimer’s disease, hypertension, cancer, and chronic lung disease), gender, age (45–54, 55–64, 65–74, and ≥75 years), marital status, education level (less than lower secondary education, upper secondary & vocational training, and tertiary education), type of medical insurance, occupation (unemployed, employed, not working, never work, retired, non-agricultural work, and agricultural work), and personal monthly consumption levels (averaged into five levels) (Table 1).

**Table 1:** Selection and description of variables.

Variable category	Variable name	Variable description
**Independent variable**	Depression level	no = 0; yes = 1
**Dependent variable**	Medical expenditure	Continuous variables
**Covariate**	Number of other chronic diseases	Continuous variables
	Consumption level	0-20% = 1;21-40% = 2; 41-60% = 3;61-80% = 4;81-100% = 5
	Demographic characteristics	
	Gender	Male = 1; Female = 2
	Age	45-55 = 1; 56–65 = 2; 66–75 = 3; 76–85 = 4; Above 86 = 5
	Marital status	Married = 1; live together = 2; Separated = 3; Divorced = 4; Never married = 5

### Statistical analyses

The following statistical analyses were performed using SPSS software: (1) general descriptive analysis, providing statistics on gender, age, marital status, education level, and occupation of middle-aged and older heart disease patients in 2018; (2) single-factor variance test, determining factors influencing medical expenditures; (3) multivariate linear regression, performing regression analysis on factors highly correlated with medical expenditures. Test level, α = 0.05.

## Results

### Prevalence of depressive symptoms and characteristics of the study sample

The study sample comprised 2445 cardiac patients, with 1229 patients (50.27%) experiencing comorbid depression and 1216 patients (49.73%) without depressive symptoms. There were 954 males (39.02%) and 1491 females (60.98%). In terms of age, 584 patients (23.89%) aged 45–55 years, 804 (32.88%) aged 56–65 years, 775 (31.7%) aged 66–75 years, 265 (10.84%) aged 66–75 years, 17 (0.7%) aged >86 years. In terms of education, 2117 patients (86.58%) completed junior high school education and below, 268 (10.9%) completed high school and vocational school, and 60 (2.45%) hold a college degree or higher. In terms of marital status, 1925 patients (78.73%) were married, 116 (4.74%) were living together, 7(0.29%) were separated, 34 (1.39%) were divorced, 355 (14.52%) were widowed, and 8 (0.33%) were unmarried. In terms of occupation, 72 patients (2.94%) were engaged in the agriculture sector, 722 (29.53%) were self-employed in the agriculture sector, 280 (11.45%) were involved in the non-agriculture sector, 108(4.42%) were self-employed in the non-agriculture sector,31(1.27%) were Unemployed,15(0.61%) were retired, 43 (1.76%) had never worked. In terms of medical insurance, 420 patients (17.18%) had purchased urban employee medical insurance, 269 (11%) had purchased urban and rural residents’ medical insurance, 145 (5.93%) had purchased urban residents’ medical insurance, 1497 (61.32%) had purchased new rural cooperative medical insurance, and 114 (4.6%) had no medical insurance (Table 2).

**Table 2:** Demographic characteristics of middle-aged and elderly patients with heart disease.

Name		Number	Percentage (%)
**Gender**	Male	954	39.02
	Female	1491	60.98
**Age**	45–55	584	23.89
	56–65	804	32.88
	66–75	775	31.7
	76–85	265	10.84
	Above 86	17	0.7
**Education level**	Lower secondary education	2117	86.58
	Upper secondary & Vocational training	268	10.96
	Tertiary education	60	2.45
**Marital status**	Married	1925	78.73
	Temporarily Separated	116	4.74
	Separated	7	0.29
	Divorced	34	1.39
	Widowed	355	14.52
	Never married	8	0.33
**Occupation**	Agricultural employed	72	2.94
	Agricultural self-employed	722	29.53
	Not agricultural employed	280	11.45
	Not agricultural self-employed	108	4.42
	Unemployed	31	1.27
	Not working	15	0.61
	Retirement	1174	48.02
	Never worked	43	1.76
**Depression level**	No	1216	49.73
	Yes	1229	50.27
**Insurance**	No insurance	114	4.6
	Urban employee medical insurance	420	17.18
	Urban and rural resident medical insurance	269	11
	Urban resident medical insurance	145	5.93
	New rural cooperative medical insurance	1497	61.32

### Influencing factors related to medical expenditures

The results of the single-factor variance test revealed statistically significant differences (p < 0.05) in medical expenditures among patients with depressive symptoms, different genders, different education levels, different numbers of other chronic diseases, and different consumption levels (Table 3).

**Table 3:** Results of one-way ANOVA test for influencing factors related to medical expenditures.

Name		Medical expenditures	F value	*P* value
**Gender**	Male	18346.06 ± 56422.53	7.73	0.005**
	Female	13577.87 ± 27734.65		
**Age**	45–55	16408.70 ± 62548.35	0.206	0.935
	56–65	15639.60 ± 32654.79		
	66–75	14777.72 ± 33746.74		
	76–85	14943.29 ± 25498.38		
	Above 86	10419.12 ± 9905.21		
**Education level**	Lower secondary education	13979.43 ± 28756.58	10.297	0.000**
	Upper secondary & vocational training	23849.36 ± 91411.65		
	Tertiary education	29344.47 ± 54815.12		
**Marital status**	Married	15438.25 ± 33359.37	0.517	0.764
	Temporarily Separated	20554.69 ± 125623.02		
	Separated	20715.71 ± 36493.36		
	Divorced	12233.70 ± 12008.26		
	Widowed	14084.43 ± 24870.02		
	Never married	10356.50 ± 12735.68		
**Occupation**	Agricultural employed	8532.15 ± 15000.55	1.321	0.236
	Agricultural self-employed	12562.35 ± 28976.77		
	Not agricultural employed	16038.23 ± 85140.80		
	Not agricultural Self-employed	14144.73 ± 29604.07		
	Unemployed	20513.29 ± 49641.72		
	Not working	10680.67 ± 11635.15		
	Retirement	17417.23 ± 33688.42		
	Never worked	18607.81 ± 27902.96		
**Depression level**	No	13094.52 ± 29645.96	7.767	0.005**
	Yes	17757.37 ± 50341.63		
**Number of other chronic diseases**	0	10788.31 ± 22638.81	3.834	0.001**
	1	13771.28 ± 33853.03		
	2	18195.52 ± 59292.87		
	3	21038.21 ± 39473.37		
	4	17716.68 ± 19344.48		
	5	19731.95 ± 13975.51		
	6	126000.00 ± null		
**Consumption level**	0–20%	9324.42 ± 9258.93	14.565	0.000**
	21–40%	9622.15 ± 22722.60		
	41–60%	10641.46 ± 19120.08		
	61–80%	17080.42 ± 33832.83		
	81–100%	25478.59 ± 70440.32		
**Insurance**	No insurance	21349.35 ± 69329.71	14.823	0.000**
	Urban employee medical insurance	14443.37 ± 27277.35		
	Urban and rural resident medical insurance	16310.85 ± 42076.67		
	Urban resident medical insurance	13866.06 ± 30934.98		
	New rural cooperative medical insurance	11011.30 ± 22251.16		

^1^ *, p < 0.05; **, p < 0.01

### Multi-factor analysis of medical expenditures

To further explore the relationship between depression level and healthcare expenditure, we conducted a multiple regression analysis with depression level as the independent variable, healthcare expenditure as the dependent variable, and control variables included influences that were shown to be statistically significant via univariate analysis, such as gender (dummy variable), level of education (dummy variable), type of healthcare insurance (dummy variable), and level of consumption. Specifically, gender, education level, type of health insurance, consumption level, and the number of other chronic diseases was significantly associated with higher healthcare expenditures (p < 0.05).The results of multiple linear regression revealed that by excluding variables without statistical significance (p > 0.05), the medical expenses of depressed patients with heart disease who were males, had completed junior high school education or below, had no medical insurance, had high consumption levels, and had many chronic diseases were higher than non-depressed patients with heart disease who were females, had completed high school or vocational school education, had urban employee medical insurance or urban residents medical insurance, had low consumption levels, and had few chronic diseases (Table 4).

**Table 4:** Multivariate analysis based on key variables affecting healthcare expenditures of middle-aged and elderly cardiac patients.

Linear regression analysis results (n = 2445)							
	Unstandardized regression coefficient		Standardized regression coefficient	t	p	Collinearity diagnostics	
	B	Standard error	Beta			VIF	TOL
**constant**	−2218.903	2423.395	–	−0.916	0.36	–	–
**Gender** **(ref: female)**							
**Male**	3661.401	1711.842	0.043	2.139	0.033*	1.055	0.948
**Education level** **(ref: upper secondary & vocational training)**							
**Lower secondary education**	7459.89	2660.871	0.056	2.804	0.005**	1.046	0.956
**Tertiary education**	9257.328	5392.276	0.035	1.717	0.086	1.053	0.95
**Insurance** **(ref: New rural cooperative medical insurance)**							
**Urban employee medical insurance**	−10402.663	2217.501	−0.095	−4.691	0.000**	1.059	0.945
**Urban and rural resident medical insurance**	676.066	2657.573	0.005	0.254	0.799	1.046	0.956
**Urban residents medical insurance**	−8204.947	3494.045	−0.047	−2.348	0.019*	1.031	0.97
**No insurance**	12536.415	3928.643	0.064	3.191	0.001**	1.028	0.973
**Consumption level**	4434.173	690.327	0.129	6.423	0.000**	1.046	0.956
**Number of other chronic diseases**	2282.621	780.052	0.058	2.926	0.003**	1.021	0.98
**Depression level**	5395.03	1662.82	0.065	3.245	0.001**	1.046	0.956
**R 2**	0.062						
**Adjusted R 2**	0.058						
**F**	F (10,2434)=16.102,p = 0.000						
**D-W**	0.294						

^2^ Dependent variable: Medical expenditure. * p < 0.05 ** p < 0.01

## Discussion

The present study used descriptive statistics to assess the presence of depressive symptoms among heart disease patients, revealing that 50.27% of patients with heart disease exhibited such symptoms. In a cross-sectional study conducted in Zhejiang Province, China, the prevalence of anxiety and depression among patients with coronary heart disease during the COVID-19 pandemic was 11.72% and 9.20%, respectively [23]. In a study carried out in rural China, 15.4% of older patients diagnosed with Huntington’s disease experienced depression [18]. The occurrence of depressive symptoms among patients with heart disease in the present study is higher than the prevalence of depression combined with heart disease in other studies [24]. This higher occurrence could be attributed to the utilization of a self-report scale, as opposed to a clinical diagnosis, for detecting depression in this study. Consequently, the study included a substantial number of participants who may not meet the formal criteria for depression but exhibit depressive symptoms in clinical settings. Nevertheless, studies [25,26] have shown that even these samples exert an influence on healthcare costs; hence, they were included in the present study.

In this study, multiple regression analysis with depression level as the independent variable and healthcare expenditure as the dependent variable demonstrated a positive correlation between depressive symptoms and healthcare expenditures. This finding indicates that healthcare costs are notably higher in cardiac patients experiencing depression than their non-depressed counterparts. Limited prior research has delved into the connection between depressive symptoms and healthcare spending in middle-aged and older cardiac patients. Most studies in this realm have focused on exploring the influence of depressive symptoms on healthcare spending among patients with other chronic conditions. An investigation into the supplementary medical costs linked to depressive symptoms in patients with chronic lung disease revealed an association between depressive symptoms and heightened medical expenses among individuals with chronic lung disease. In the study, increased medical expenses attributed to depressive symptoms rose by 20.0% among patients exhibiting mild to moderate symptoms and by 69.2% among those experiencing severe depressive symptoms [27]. An additional investigation into the economic burden of depressive symptoms among patients with hypertension revealed an association between depressive symptoms and an increased direct economic burden in middle-aged and older patients with hypertension. Financial losses were found to be positively correlated with depression levels [28]. An investigation into medical expenditures related to depressive symptoms among older patients in rural China demonstrated that compared to other demographic groups, mental health conditions exerted a significant effect on individual medical expenses, rural populations, females, widowed individuals, and those with lower levels of education exhibited higher medical expenditures attributable to depressive states [29]. Consistently, these studies demonstrate that depressive symptoms lead to increased healthcare expenditures among patients with chronic diseases. In addition, a Swedish study found that depressive symptoms have no significant effect on the medical expenses of patients with back pain, which may be because diverse types of diseases need distinct healthcare services; among patients experiencing back pain, a substantial portion of their drug treatments overlap, resulting in anticipated reductions in drug costs [30].

The present study draws the following conclusions. First, extensive evidence has established depression as a risk factor for heart disease, and it can exacerbate patients’ conditions, contributing to increased morbidity, readmissions, mortality, and deterioration of medical outcomes [31], thereby resulting in elevated medical expenditures. Regarding endogenous mechanisms, depression stands as an independent risk factor contributing to the decline of patients with heart disease [32]. Several studies have indicated that depression may induce alterations in the autonomic nervous system, influence the gut microbiome, and elevate inflammation levels, contributing to the development of heart disease [33]. Besides,depression often coexists with other emotional distress [34], a study indicated that more than half the patients with anxiety also met criteria for clinical depression,it further leads to the development of the patient’s condition. In the study,it indicated that depression symptoms were associated with a 2-fold increased risk of mortality in CHD patients,but depressed patients with comorbid anxiety have a 3-fold increased risk [35]. Considering external factors, the presence of depression is associated with a decline in the patient’s self-care ability, unhealthy dietary habits, insufficient physical activity, smoking, and other adverse behaviors, deteriorating the patient’s condition [36]. Meanwhile, individuals with comorbid depression exhibit lower adherence; they may not adhere to the prescribed medication regimen as instructed by their healthcare providers, neglect recommended secondary prevention measures, and communicate less efficiently with healthcare providers, thereby affecting the effectiveness of treatment.

Second, evidence suggests an association between depression and increased healthcare utilization,depleting healthcare resources and escalating healthcare expenditures. The presence of depressive symptoms can induce panic in patients, leading to somatization, amplification of symptoms, and an intensified sense of self [37]. Patients often seek healthcare services excessively, driven by a desire for psychological comfort and to prevent the recurrence of the disease [38], which is counterproductive to the treatment of this condition. Patients with depression face an elevated risk of hospitalization; hospital stays for individuals with depression tend to be prolonged compared to the average patient, consequently heightening the risk of infection and medication side effects [39].

Finally, the current management of depression in patients with heart disease has not garnered sufficient attention in China.Screening and treatment for depression are not mandatory in China’s heart disease treatment guidelines, but only as an indicator,making it challenging for patients to access professional treatment. This issue undoubtedly aggravates the detrimental effects of depression on patients and contributes to an escalation in their medical expenses. Several studies have indicated that a substantial portion of individuals with comorbid depression frequently opt to seek assistance from primary care providers instead of specialized mental health agencies [40]. Consequently, some patients with depression do not receive appropriate treatment. Collaborative care interventions lead to an improvement in depressive symptoms among patients with heart disease when compared to those receiving standard primary care. Patients currently undergoing treatment predominantly rely on prescription drugs rather than psychotherapy [41], which not only increases the patient’s medication costs but also complicates treatment regimens and care regimens, increasing the likelihood of complications and further escalating healthcare expenditures.

The present study holds the advantage of a substantial sample size derived from household questionnaires, encompassing a diverse range of groups, ensuring its representative nature. Furthermore, this study considered confounding factors, including other chronic diseases and consumption levels, thereby bolstering the credibility of the results. Nevertheless, this study possesses certain limitations. First, the r2 value in the used model was only 0.058, indicating a poor fit. For comparison, a Chinese study investigating the relationship between water quality and depression reported an r2 value of 0.069 [42]. In a prospective study investigating the mediation of emotional eating in the association between depression and 7-year changes in body mass index and waist circumference, the r2 value was 0.048 [43]. The specificity of depression as a psychiatric factor may account for the low r^2^ value in our study. However, as the model in our study primarily investigates correlations instead of making predictions, its effect on the results of this study is limited. Second, this study relied on self-reporting through the short version of the Depression Measurement Scale, a factor that may introduce mismeasurement and pose challenges in accurately determining the true prevalence. Third, this cross-sectional study did not observe the influence of depressive symptoms on healthcare expenditures over the long term, potentially leading to confounding by other extraneous factors. Fourth, the medical expenditure data primarily relied on subjects’ memory, introducing the possibility of recall bias. Finally, since this paper is a cross-sectional study, it proves the correlation between depression and medical expenditure in patients with heart disease, but it cannot infer the causal relationship between depressive symptoms and medical expenditure

In conclusion, the findings of this study indicate a positive association between depressive symptoms and medical expenditures in older patients with heart disease. Although depression can lead to increased morbidity and mortality, the current treatment of middle-aged and older cardiac patients does not emphasize the treatment of depression. In the future, it is advisable to enhance the provision of mental health care services for middle-aged and older patients with heart disease, along with increasing collaborative care interventions specific to heart disease.
